# The Psychometric Properties of the Persian Version of Childhood Experience of Care and Abuse Questionnaire (CECA)

**DOI:** 10.31661/gmj.v9i0.1663

**Published:** 2020-06-23

**Authors:** Peymaneh Shirinbayan, Mahyar Salavati, Farin Soleimani, Ahmad Saeedi, Mohammad Asghari-Jafarabadi, Sahel Hemmati, Roshanak Vameghi

**Affiliations:** ^1^Pediatric Neurorehabilitation Research Center, University of Social Welfare and Rehabilitation Sciences, Tehran, Iran; ^2^Department of Physiotherapy, University of Social Welfare and Rehabilitation Sciences, Tehran, Iran; ^3^Institute for Research and Planning in Higher Education, Tehran, Iran; ^4^Road Traffic Injury Research Center, Tabriz University of Medical Sciences, Tabriz, Iran; ^5^Department of Statistics and Epidemiology, Faculty of Health, Tabriz University of Medical Sciences, Tabriz, Iran

**Keywords:** Adolescent, Validation, Neglect, Child Abuse

## Abstract

**Background:**

The present study aimed to determine the psychometric properties of the Persian version of the Childhood Experience of Care and Abuse Questionnaire (CECA.Q), a tool based on a retrospective interview with the child.

**Materials and Methods:**

To this aim, 251 adolescents from four regions of Tehran megacity completed the questionnaire. The reliability of the questionnaire was examined, along with the face and content validity. In addition, the construct validity was evaluated by exploratory factor analysis (EFA) and confirmatory factor analysis (CFA).

**Results:**

EFA and CFA supported a 4-factor solution including mother’s role scale items, father’s role scale items, maternal behavior scale items, and paternal behavior scale items. The total variance extracted in EFA ranged from 33.9 to 60.7. The internal consistency for mother’s role, father’s role, maternal behavior, and paternal behavior was 0.61, 0.65, 0.86, and 0.9 respectively. Thus, the questionnaire had a suitable fit, as well as reasonable reliability and validity.

**Conclusion:**

The Persian version of the CECA.Q had adequate reliability and validity as a self-report measurement for childhood experience of care and abuse.

## Introduction

It is believed that certain parental behaviors and attitudes predispose the child to psychiatric disorders, along with dysfunctional social and emotional behaviors in adulthood [1]. Some evidence indicated that harmful childhood events and experiences such as loss of a parent, family dysfunction, neglect, antipathy, physical abuse, and sexual abuse increase the risk of major depression in adulthood [2-4]. The prevalence of childhood physical and emotional abuse is certainly related to depressive symptoms [5], which has a lifelong outcome on the overall mental health [6]. A large number of some studies indicated that retrospective interviews regarding childhood experiences are eligible and can be satisfactorily applied [7]. Childhood Experience of Care and Abuse Questionnaire (CECA.Q) is regarded as one of the instruments developed based on such interviews, which includes childhood experiences before the age of 17. The psychometric properties of this instrument were satisfactory in other communities [8] and the relationship of the scale to depression was demonstrated in community samples [9]. Further, CECA.Q is considered as a semi-structured retrospective questionnaire of childhood experiences, designed to be answered by adults and adolescents, which focuses on the course of life before the age of 17. Conducting the interview takes about 40-120 minutes based on how complex and intricate the childhood experiences [9]. Further, the tool seems to be appropriate for use as a screening test [10]. Although the instrument has been already validated in communities different from the Iranian adolescent community in social and cultural terms, the present study aimed to reassess the psychometrics of the Persian version of this tool in a representative sample of the healthy Iranian adolescent population.

## Materials and Methods

###  Participants

 A total of 251 children, aged 11-14, were randomly selected by multistage cluster sampling method in Tehran from June to September 2018. The sampling was performed in Northern, Southern, Eastern, and Western regions of Tehran for the purpose of covering various cultural and socioeconomic classes. The number of samples recruited from each region was calculated with respect to the population size in each region, school areas, café shops, cinemas, sports clubs, parks, libraries. In addition, the internet cafés were the places the adolescents (girls and boys) were selected for the study. In this study, only psychologically normal adolescents were included, while those already diagnosed with psychological disorders were excluded. For this purpose, we asked the adolescent and his/her mother whether he/she had a history of psychiatric hospitalization, taking psychiatric medications or being under the supervision of a psychiatrist, and if so, he/she was excluded from the study.

###  Procedure 

 Permission was obtained from the original author of the CECA.Q. The study was confirmed by Ethics Committee of the University of Social Welfare and Rehabilitation Sciences (ID: IR.USWR.REC.1398.008). Informed consent was obtained from all of the participants included in the study, as well as their parents. The CECA.Q was translated into Persian by two independent translators. Then, both translators discussed any difference in their translations and agreed upon a single translated version. The backward translation to English was performed by another professional translator with the experience of living in English-speaking countries. In the next procedure, the back-translated version was scrutinized for any possible incongruity with the original English version of the CECA.Q and the necessary corrections were made in the Persian version by the research team. Then, the face validity of the final translated version was evaluated qualitatively. To this aim, 20 adolescents were recruited by convenient sampling method from four different regions in Tehran with the same inclusion and exclusion criteria as the study sample. In addition, they were interviewed to detect any possible ambiguities or misunderstandings or other forms of difficulties in completing the questionnaire. First, the project and its objectives were explained for adolescents and their parents in person or by phone. The consent form was signed after agreement. Next, a suitable rapport was built up with adolescents in order to gain their trust in providing the correct answers to the items of the questionnaire, which was sent to the adolescents’ homes to be completed. The researcher contacted the participants in order to ensure that the questionnaires were completed correctly. Further, the researcher’s contact number was provided for the samples for any questions or ambiguity in completing the questionnaire. The participants who left questions unanswered or decided not to continue participating were excluded from the study. In addition to the CECA.Q, some demographic data including gender, age, and geographic region of residence, parental education, and occupation were obtained.

###  Contents of the Questionnaire and Scoring

The CECA.Q covers four main areas including “neglect,” involving parents’ disregard in terms of maternal care (feeding and clothing), health, schoolwork, and friendships of the child, “antipathy,” which includes animosity, disregard, rejection or ‘scapegoating’ behavior shown to the child by parents, “physical abuse” such as being struck by parents or other older domestic members, and ”sexual abuse,” or physical contact or coming close to the child of a sexual nature by an adult [9]. The four main mentioned areas were covered in three subscales including parental care (neglect and antipathy), physical abuse, and sexual abuse. The scoring is done online on the CECA.Q website [11]. CECA.Q parental care: A total of 16 items cover a mixture of antipathy (8 items) and neglect (8 items) from mother and father. Scoring is done on a 5-point scale (1=deﬁnitely yes, 3=unsure, 5=not at all). Answers to these questions identify the connection to the caregiver. Regarding scoring mother antipathy, the scores of items 8, 9, 10, and 11 are reversed. Then, the items 1, 4, 6, 8, 9, 10, 11, and 16 are added. In the next procedure, the same procedure is repeated to calculate the father’s antipathy score. As for scoring mother’s neglect, the scores of items 2, 3, 5, 12, and 14 are reversed. Then, the scores of items 2, 3, 5, 7, 12, 13, 14, and 15 are added. Finally, the same procedure is repeated to calculate father’s neglect score [10]. CECA.Q physical abuse: This subscale covers physical punishment by the parent or other family members. The physical abuse subscale starts by asking the child or teenager “were you ever hit repeatedly with an implement such as belt or stick, or pierced, kicked, or burnt by someone in the household?”. If the answer is “yes,” some questions are asked to define further details of the physical abuse by the mother or the father such as “When was the ﬁrst physical punishment or abuse done, “Did it occur on more than one occasion?”, “How was the child physically punished (belt/stick, punched/kicked, hit with hand, or other forms)?”, “Did any hurt and harm such as bruises, black eyes, or broken limbs occur?”, and “Was the punishing mother or father out of control?”. For scoring, item scores are summed up [10]. CECA.Q sexual abuse: In this subscale, three questions including unwanted sexual experiences, sexual intercourse against your request, and sexual experiences with a relevant adult or someone else were asked. “Yes” and “unsure” were considered as positive responses for scoring targets.

###  Statistical Analyses

The Cronbach’ alpha coefficient and Intraclass Correlation Coefficient (ICC) were determined to assess the internal consistency and reliability of the scale, respectively. The values of ≥0.70 were considered as sufficient, 0.60–0.70 as medium, and 0.50–0.60 as poor, and those smaller than 0.50 were considered unacceptable [12]. In addition, exploratory factor analysis (EFA) was used to see the possible factor solutions. EFA was conducted by Principal Axis Factoring (PAF) Further, varimax rotation with Kaiser Normalization was used for extraction method. The scree plot procedure was used to decide the number of the factors which should be extracted. The KMO (Kaiser-Meyer-Olkin) measure of sampling adequacy and Bartlett’s test of sphericity were used to evaluate the sufficiency of the model. It is generally accepted that high values of KMO (more than 0.7) suggest that factor analysis may be useful with the existing data. Bartlett’s test of sphericity was used to test the hypothesis that a correlation matrix is an identity matrix, which would, in turn, demonstrate that variables are unrelated and thus inappropriate for structure detection. The significance values less than 0.05 indicate a satisfactory factor analysis. Furthermore, the total variance explained was reported. Factor loading values of 0.3 or higher were considered to indicate a relationship between items and factors. The construct validity was assessed using conﬁrmatory factor analysis (CFA) by using the method of weighted least squares with a weighed matrix of asymptomatic covariance for estimation. Fit indices and reasonable values of these indices for CFA were considered as χ2/df<5, Root Mean Square Error of Approximation (RMSEA)<0.08, and also the Comparative Fit Index (CFI), Goodness of Fit Index (GFI), and Adjusted Goodness of Fit Index (AGFI)>0.9 [13]. Data analysis was performed in SPSS 17.0 (SPSS Inc., Chicago, IL, USA) and AMOS16 (SPSS Inc., Chicago, IL, USA) for CFA. P-values less than 0.05 were considered as significant.

## Results

###  Face Validity

 No change was made in the content and structure of the questionnaire since no problem or ambiguity were detected on completing the questionnaire by a group of 20 adolescents recruited for the purpose of determining the face validity of the tool.

###  Reliability

 As shown in Table-1, the Cronbach α coefficients for the mother and father care scale items of the CECA.Q were 0.611 and 0.651, respectively, which reflects appropriate reliability (r>0.7). Regarding mother physical abuse items and father physical abuse, the coefficients were 0.866 and 0.900, respectively, which confirmed the reliability of the scale.

###  Construct Validity

####  Exploratory Factor Analysis (EFA)

 As shown in Table-2, the Kaser-Meier-Olkin (KMO) value for the mother care subscale items was 0.824 (0.53 to 0.73), ranged from acceptable levels to desirable levels for this index. The total variance of the scale was explained between 33.9 and 60.7. The Bartlett’s test of sphericity indicated the P-value of <0.05, which is consistent with the KMOs and confirmed the adequacy of EFA for all studied constructs. Furthermore, the factor scales higher than 0.3 (criteria for item selection) were observed in all subscales, which confirmed the relationship between this construct and the whole concept of the questionnaire, except in items including the sexual abuse subscale. Based on the results, the EFA for all subscales had a fair level of adequacy (KMO>0.7, Bartlett’s value <0.05). Finally, an acceptable variance of the subscales was explained by the extracted factors, ranged 33.9-60.6%.

###  Confirmatory Factor Analyses for the CECA-Q

####  Model Adequacy 

Table-3 indicates the fit indices for evaluating the appropriateness of CFA for the mother care subscale. Based on the values of the presented indexes for all subscales, the Chi-square index over its degree of freedom was smaller than 5, and the values of RMSEA and RMR were smaller than 0.08 and 0.1, respectively, which confirm the adequacy of the model. In addition, GFI, AGFI, NFI, NNFI, RFI, IFI, and CFI fit indices were bigger than 0.9. As a result, these models achieved an efficient level of fitness, confirming the factor structure.

###  Path Diagram with Standard Coefficients for the CECA.Q 

**Figure 1:**
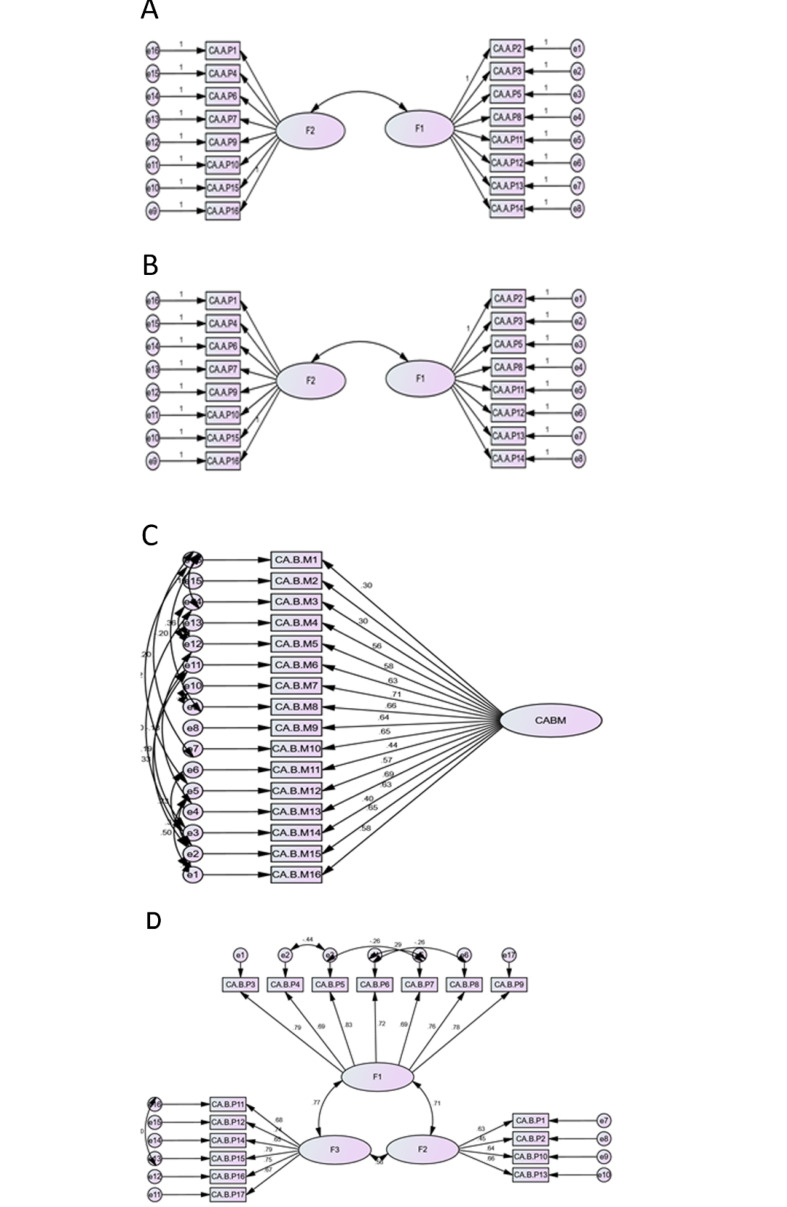
A) mother care subscale, B) father care subscale, C) mother physical abuse subscale and D) father physical abuse subscale

 The standard coefficients of the CFA for this questionnaire are demonstrated in Figures-1A-D. Path diagram of the CECA.Q reveals the standardized parameters relating items to CECA.Q. In fact, all parameters were statistically significant (P<0.05) (Figures-1A-D).

###  One-factor EFA Result for Mother Care Scale 

 Factor Matrix

Table-4 presents a successful single-factor solution extracted by EFA in the exploratory step.

###  Two-factor Structure for Father Care Scale

 A successful two-factor solution extracted by EFA in the exploratory step is demonstrated in Table-5.

###  One-factor Structure for Mother Physical Abuse Scale

 Factor Matrix

Table-6 indicates a successful one-factor solution extracted by EFA in the exploratory step.

###  Three-factor Structure for Father Physical Abuse Scale

 Factor Matrix

 As shown in Table-7, a successful three-factor solution is extracted by EFA in the exploratory step.

## Discussion

CECA.Q is a useful instrument in screening experiences of care and abuse in childhood and for detecting the danger of childhood depressive disorder. Experiencing abuse in childhood is strongly related to emotional inconsistency in adulthood (5). Physical and emotional abuse signiﬁcantly reduces dutiful and expanded receptivity, while the relationship between physical abuse and receptivity is not yet clear. It is worth noting that high receptivity can affect impulsive and aggressive desire (14). In childhood, sexual abuse is related to depressive symptoms and the results of the previous studies could support that childhood abuse has an enduring effect on mental health in life (6). The present study evaluated the psychometric properties of the CECA.Q among a sample of Iranian adolescents living in Tehran, Iran. The results demonstrated desirable reliability and validity for the CECA.Q. The alpha coefficients for antipathy and neglect as the parental care were 0.611 and 0.651, which indicate medium internal consistency. Based on the results of factor analysis, KMO value above 0.7 (0.824) could confirms the adequacy of the factor model. The results of the variance explained by each factor and the total variance showed that 33.94% of variances were explained by one extracted factor. The findings are consistent with those of the previous studies which assessed the reliability and validity of the CECA.Q [10,11]. Bifulco *et al*. evaluated the psychometric properties of CECA.Q in London and indicated sufficient internal consistency for antipathy (α=0.81), and neglect (α=0.80) in mother’s physical abuse (α=0.52), and father’s physical abuse (α=0.51) [10]. In the present study, the Cronbach α value for mother’s care (antipathy & neglect) (α=0.611), father’s care (antipathy & neglect) (α=0.651), mother’s physical abuse (α=0.866), and father’s physical abuse (α=0.900) approved the high internal consistency of the questionnaire.

## Conclusion

 Regarding the analyses conducted for the maternity care, father care, maternal behavior, and paternal behavior subscales, the CFA possessed relatively appropriate fitness in three of the factors and the results confirmed a significant relationship among the scale items. The findings of the exploratory factor model were supported by the confirmatory patterns. In addition, the structural reliability of the scale was confirmed in three factors. Consequently, the Persian version of the CECA.Q has acceptable psychometric properties and can be used in research and clinical practice, except for the questions regarding sexual abuse which may need to be modified for the Iranian population.

## Acknowledgment

 The authors thank all parents who participated in this study. The study was funded by a grant from Iran National Science Foundation (INSF, number: 947025111) for Ph.D. dissertation.

## Conflict of Interest

 The authors declare that they have no conflict of interest.

**Table 1:** The Internal Consistency (the Cronbach α coefficients) for the Persian Version of the CECA.Q in Iranian Adolescents

**CECA.Q subscale**	**The Cronbach α coefficient (N=251)**
Mother care scale items	0.61
Father care scale items	0.65
Mother physical abuse items	0.87
Father physical abuse items	0.90

**Table 2:** Results of KMO, Bartlett’s Test of Sphericity, and Total Variance Explained

**Subscales**	**KMO**	**Bartlett’s test of sphericity**	**Total variance explained**
Mother Care	0.82	Chi2(120)=1117.7, P<0.001	33.9
Father Care	0.84	Chi2(120)=964.1, P<0.001	46.7
Mother physical abuse	0.86	Chi2(120)=1519.1, P<0.001	38.9
Father physical abuse	0.87	Chi2(136)=1995.4, P<0.001	60.7

**KMO:** Kaiser-Meyer-Olkin measure of sampling adequacy

**Table 3:** CFA Fit Indices for the Scales of CECA.Q

**Subscales**	**χ 2**	**df**	**P**	**χ 2/ df**	**RMR**	**GFI**	**AGFI**	**NFI**	**RFI**	**IFI**	**NNFI**	**CFI**	**RMSEA (95%CI)**
Mother Care	237.40	94	<0.001	2.53	0.038	0.98	0.93	0.91	0.96	0.98	0.94	0.98	0.078 (0.065; 0.090)
Father Care	209.24	100	<0.001	2.09	0.038	0.91	0.90	0.95	0.92	0.99	0.95	0.99	0.066 (0.053; 0.078)
Mother physical abuse	223.93	89	<0.001	2.52	0.038	0.98	0.95	0.92	0.93	0.92	0.93	0.96	0.077 (0.065; 0.090)
Father physical abuse	360.93	111	<0.001	3.25	0.038	0.97	0.92	0.94	0.91	0.99	0.96	0.99	0.074 (0.064; 0.095)

**Abbreviations: **χ2, Chi-square; df, degrees of freedom; χ2/df, normed Chi-square; **RMR:** Root Mean R; **GFI: **Goodness of Fit Index; **AGFI:** Adjusted Goodness of Fit Index; RMSEA, root mean square error of approximation; **NFI: **Normed Fit Index; **RFI: **Relative Fit Index; **IFI:** Incremental Fit Index; **NNFI: **Non-Normed Fit Index; CFI, comparative fit index

**Table 4:** Loading of Rotated Factor Matrix for Mother Care Subscale

	**Factor Matrix**	**Factor 1**
**1**	At times, she made me feel I was a nuisance.	0.70
**2**	She often picked on me unfairly.	0.68
**3**	She was concerned about my whereabouts.	-0.67
**4**	She would leave me unsupervised before I was 10.	0.64
**5**	She did not like me as much as my brothers and sisters (Leave blank if no siblings).	0.62
**6**	She was there if I needed her.	-0.56
**7**	She would usually have time to talk to me.	-0.54
**8**	She cared for me when I was ill.	-0.53
**9**	She made me feel unwanted.	0.53
**10**	She was interested in knowing who my friends were.	-0.53
**11**	She was interested in how I did at school.	-0.52
**12**	She neglected my basic needs (e.g. food and clothes).	0.51
**13**	She was very difficult to please.	0.47
**14**	She was concerned about my worries.	-0.45
**15**	He tried to make me feel better when I was upset.	-0.40
**16**	She was very critical of me.	0.30

**Extraction method:** Principal axis factoring.

**Table 5:** Loading of Rotated Factor Matrix for Father Care Subscale

	**Factor Matrix**	Factor	
		1	2
**1**	At times, he made me feel I was a nuisance.	0.81	
**2**	He often picked on me unfairly.	0.76	
**3**	He made me feel unwanted.	0.72	
**4**	He would leave me unsupervised before I was 10.	0.66	
**5**	He did not like me as much as my brothers and sisters	0.55	
**6**	He neglected my basic needs (e.g. food and clothes).	0.54	
**7**	He was very difficult to please.	0.50	
**8**	He was very critical of me.	0.46	
**9**	He would usually have time to talk to me.		0.73
**10**	He tried to make me feel better when I was upset.		0.63
**11**	He cared for me when I was ill.		0.61
**12**	He was interested in how I did at school.		0.58
**13**	He was there if I needed him.		0.57
**14**	He was concerned about my whereabouts.		0.55
**15**	He was interested in knowing who my friends were.		0.51
**16**	He was concerned about my worries.		0.50

**Extraction method: **Principal axis factoring. **Rotation method: **Oblimin with Kaiser Normalization.

**Table 6:** Loading of the Rotated Factor Matrix

**Factor Matrix of physical abuse**	Factor 1
MP6	0.73
MP 15	0.73
MP 9	0.70
MP 12	0.68
MP 5	0.67
MP 7	0.65
MP 8	0.62
MP 16	0.62
MP 3	0.62
MP 14	0.60
MP 11	0.59
MP 4	0.58
MP 10	0.44
MP 13	0.39
MP 2	0.33
MP 1	0.32
MP 17	0.32

**MP: **Mother physical abuse, **Extraction method:** Principal axis factoring.
